# Role of high-dose exposure in transmission hot zones as a driver of SARS-CoV-2 dynamics

**DOI:** 10.1098/rsif.2020.0916

**Published:** 2021-03-31

**Authors:** Dominik Wodarz, Natalia L. Komarova, Luis M. Schang

**Affiliations:** ^1^ Department of Population Health and Disease Prevention, Program in Public Health, Susan and Henry Samueli College of Health Science, University of California Irvine, Irvine, CA 92697, USA; ^2^ Department of Mathematics, University of California Irvine, Irvine, CA 92697, USA; ^3^ Department of Microbiology and Immunology and Baker Institute, Cornell University College of Veterinary Medicine, Cornell University, Ithaca, NY 14853, USA

**Keywords:** SARS-CoV-2, COVID-19, epidemiology, infectious dose

## Abstract

Epidemiological data about SARS-CoV-2 spread indicate that the virus is not transmitted uniformly in the population. The transmission tends to be more effective in select settings that involve exposure to relatively high viral dose, such as in crowded indoor settings, assisted living facilities, prisons or food processing plants. To explore the effect on infection dynamics, we describe a new mathematical model where transmission can occur (i) in the community at large, characterized by low-dose exposure and mostly mild disease, and (ii) in so-called transmission hot zones, characterized by high-dose exposure that can be associated with more severe disease. The model yields different types of epidemiological dynamics, depending on the relative importance of hot zone and community transmission. Interesting dynamics occur if the rate of virus release/deposition from severely infected people is larger than that of mildly infected individuals. Under this assumption, we find that successful infection spread can hinge upon high-dose hot zone transmission, yet the majority of infections are predicted to occur in the community at large with mild disease. In this regime, residual hot zone transmission can account for continued virus spread during community lockdowns, and the suppression of hot zones after community interventions are relaxed can cause a prolonged lack of infection resurgence following the reopening of society. This gives rise to the notion that targeted interventions specifically reducing virus transmission in the hot zones have the potential to suppress overall infection spread, including in the community at large. Epidemiological trends in the USA and Europe are interpreted in light of this model.

## 1. Introduction

As the United States and other countries around the world have witnessed waves of SARS-CoV-2 spread and the associated morbidity and mortality, it is clear that a better understanding of SARS-CoV infection dynamics will benefit the efforts to reduce infection burden, through non-pharmaceutical interventions and vaccines. Mathematical models have been used to characterize the dynamics of SARS-CoV-2 and predict potential numbers of COVID-19 cases [1–7], which has resulted in the estimation of the basic reproduction number [1,8], a better understanding of expected transmission dynamics in the absence and presence of non-pharmaceutical interventions [9–16], and in the critical effect of age structure on disease dynamics [11,17], among many other contributions. Some of these models have been extremely useful for predicting and quantifying the demands on healthcare resources.

At the same time, it is becoming clear that the spread of SARS-CoV-2 is characterized by unique aspects that have so far not been fully captured by the epidemiological models and that might be crucial for predicting how the virus and the disease may spread depending on the degree to which the society and economy is open. Epidemiological data on infection spread indicate that the virus does not spread uniformly in the population, but that some settings contribute more to virus transmission than others [18,19]. For example, it appears that outbreaks in many locations may have been driven by infection spread in select settings, such as food processing plants [20], prisons [21], assisted living facilities [22] or hospitals [23], where a group of people meets and interacts repeatedly. Infection is further promoted by several other settings where larger groups of people gather in the absence of sufficient ventilation or in close proximity without face protection, such as restaurants, bars, gyms or large gatherings/parties. Some of these settings have also been referred to as superspreading events [24,25]. These observations indicate that overall virus transmission appears to occur mostly in settings in which people are exposed to relatively high doses of the virus, and less so in the community at large, where viral doses tend to be lower. Viral dose upon exposure can influence the chances of developing a productive infection and can impact the severity of the disease. The viral infectious dose has drastic consequences for SARS and MERS infections [26,27] and for the pathogenesis of SARS-CoV-2 in animal models [28,29]. Recent evidence strongly suggests that reducing viral infection load by using facemasks has a pronounced effect on the outcome of human infections [30,31].

We collectively refer to the settings in which infection with SARS-CoV-2 is promoted through exposure to higher viral doses as ‘transmission hot zones', and contrast this with the less effective virus transmission in the community at large, characterized by exposure to a lower viral dose. Understanding the effects of hot zone transmission on infection dynamics requires the incorporation of these assumptions into mathematical models.

Here, we create a mathematical model that is based on the well-established SIR model, but it incorporates the notions of (i) asymptomatic or mild versus severe disease, (ii) the effect of exposure dose on disease severity and transmission risk and (iii) community transmission versus hot zone transmission. These concepts have already been introduced and discussed in the literature. Here, we create a computational and analytical tool which takes these notions beyond verbal reasoning and finds the logical consequences of the assumptions. Under certain assumptions, the model can give rise to several different outcomes, both during natural infection growth and during non-pharmaceutical interventions, which cannot be accounted for by simpler SIR models that lack added complexity. Among the outcomes, we find that, somewhat counterintuitively, successful infection spread can hinge under certain assumptions upon hot zone transmissions that promote severe infections, yet at the same time the majority of the SARS-CoV-2 infections are mild and occur in the community at large. The model further predicts that in such cases, targeted interventions that limit virus spread in hot zones can result in the long-term suppression of infection levels in the community at large, even if non-pharmaceutical interventions in the community are relaxed to some extent. According to our mathematical model, these dynamics are a direct consequence of the assumed viral dose-dependency, which might thus warrant further attention from a clinical and epidemiological perspective. With this theory in mind, we interpret epidemiological data that document SARS-CoV-2 dynamics in different states in the USA and in European countries.

## 2. Results

### 2.1. The mathematical modelling framework

**Figure 1.**
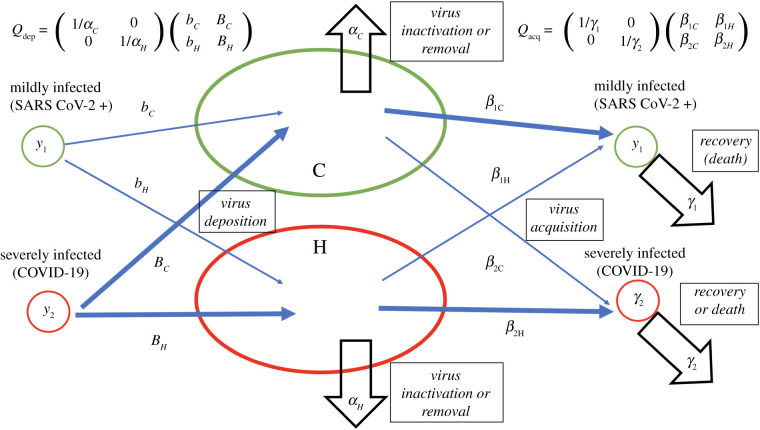
A schematic showing the model structure and its parameters. Infection deposition and acquisition matrices *Q*_dep_ and *Q*_acq_ are defined; see electronic supplementary material for details. In the model, transmission can result in two types of infected populations: asymptomatic/mild SARS-CoV-2 infection (denoted by *y*_1_) and severely SARS-CoV-2 infected, including ambulatory symptomatic COVID-19 patients (denoted by *y*_2_). It is assumed that severe COVID-19 is promoted by exposure to a higher viral load. Hence, the exposure of susceptible individuals to virus in the community at large compartment (C) results mostly in mild infection with a probability *β*_1C_, and less frequently in severe infection with a probability *β*_2C_, where *β*_1C_ > *β*_2C_ because local viral load is assumed to be relatively low in the C compartment. Exposure in the hot zone compartment (H) results in mild infection with a probability *β*_1H_, and in more frequent severe COVID-19 with a rate *β*_2H_, where *β*_1H_ < *β*_2H_, because virus load is assumed to be higher in the hot zone. Mildly infected individuals are assumed to deposit virus in the C and H compartments with rates *b*_*C*_ and *b*_*H*_, respectively. Severely SARS-CoV-2 infected, including symptomatic but ambulatory, individuals are assumed to deposit virus in those compartments at rates *B*_*C*_ and *B*_*H*_, respectively. Finally, virus decays in the two locations at rates *α*_*C*_ and *α*_*H*_, and mildly and severely infected individuals cease to be infectious (because of recovery or death) with rates *γ*_1_ and *γ*_2_.

We consider a mathematical model that distinguishes between patients with a mild or asymptomatic infection and those with severe SARS-CoV-2 infection, including symptomatic but ambulatory COVID-19 patients. We further assume that a higher infectious dose promotes the development of more severe outcomes, as has been documented with SARS, MERS and even SARS-CoV-2 [26–28,32]. In particular, the model couples viral load to the setting in which transmission takes place. Hence, we distinguish between two basic types of transmission varying in the viral load to which susceptible individuals are exposed (figure 1). The first type of transmission occurs in the ‘community-at-large’. A characteristic of this environment is that people are exposed to relatively low viral loads for short times, and that disease tends to be mild. This can include streets and other outdoor areas, as well as indoor locations where it is unlikely that several infected individuals converge periodically for long periods, and where human density is low and contacts between individuals are short and occasional. The second type of transmission is in what we call ‘hot zones’. These are characterized by exposure to high viral loads and by a higher chance of severe disease. This can result from exposure to multiple infected individuals, exposure to individuals shedding high viral levels in locations with poor ventilation, or from an increase in viral load over time through silent amplification rounds of infection [33]. The model is based on the general SIR framework [34,35]. The corresponding ordinary differential equations are given as follows:

$$\begin{array}{rl} \dot{x} & =-x[(\beta_{1H}+\beta_{2H})H+(\beta_{1C}+\beta_{2C})C],\\ {\dot{y}}_{1} & =x(\beta_{1C}C+\beta_{1H}H)-\gamma_{1}y_{1},\\ {\dot{y}}_{2} & =x(\beta_{2C}C+\beta_{2H}H)-\gamma_{2}y_{2},\\ \dot{z} & =\gamma_{1}y_{1}+\gamma_{2}y_{2},\\ \dot{C} & =b_{Cy_{1}}+B_{Cy_{2}}-\alpha_{C}C\\ \mathrm{and}\;\dot{H} & =b_{Hy_{1}}+B_{Hy_{2}}-\alpha_{H}H, \end{array}\;\;$$

where *x* denotes the population of susceptible individuals, *y*_1_ and *y*_2_ denote the populations of mildly and severely infected individuals, respectively, and *z* represents the population of removed infected (recovered and dead). Further, *C* and *H* represent environmental viral load in the community at large and in the hot zones, respectively. The processes underlying the model are further explained in figure 1, where all the parameters are defined. The following sections discuss results arising from this model, and further mathematical details are provided in the electronic supplementary material.

### 2.2. The basic reproduction number and maintenance of infection spread

We use this model to calculate the basic reproduction number of the infection, *R*_0_, as well as the effective reproduction number, *R*. We start by defining two matrices, a virus deposition matrix, *Q*_dep_, and a virus acquisition matrix, *Q*_acq_. The former matrix is given by

$$Q_{\mathrm{dep}}=\left(\begin{array}{cc} 1/\alpha_{C} & 0\\ 0 & 1/\alpha_{H} \end{array}\right)\left(\begin{array}{cc} b_{C} & B_{C}\\ b_{H} & B_{H} \end{array}\right),$$

and depends on the rates at which mildly and severely infected individuals deposit the virus both in hot zones and the community, and also on the virus lifespan in each environment. The latter matrix is defined as

$$Q_{\mathrm{acq}}=\left(\begin{array}{cc} 1/\gamma_{1} & 0\\ 0 & 1/\gamma_{2} \end{array}\right)\left(\begin{array}{cc} \beta_{1C} & \beta_{1H}\\ \beta_{2C} & \beta_{2H} \end{array}\right),$$

and depends on the infectivity coefficients for mildly and severely infected individuals in both environments, as well as the expected disease duration for mild and severely infected patients. To determine the reproductive number (including the basic reproductive number) of the infection, we form a product of the acquisition and deposition matrices,

2.1 $$A=\left(\begin{array}{cc} A_{11} & A_{12}\\ A_{21} & A_{22} \end{array}\right)=Q_{\mathrm{acq}}Q_{\mathrm{dep}}.$$

The elements of this matrix, *A_ij_*, are combinations of all of the rates (figure 1 and the electronic supplementary material). These four quantities have a clear meaning, as they express the intensity of infection spread via four pathways: *A*_11_ is the probability to become mildly infected as a result of another mildly infected individual depositing virus in a C or an H location; *A*_12_ is the probability to become mildly infected as a result of a severely infected individual depositing virus in a C or an H location, etc.

Using the matrix *A*, the basic reproductive number of the infection can be expressed concisely as

$$R_{0}=x_{0}r,$$

where *x* is the initial number of susceptible individuals and *r* is the larger of the two eigenvalues of the matrix *A*. Similarly, the effective reproductive number, *R*, is calculated as *R* = *xr*, where *x* is the current number of susceptible individuals. The quantity *r* depends on the model coefficients and will be affected, for example, by social distancing measures.

Depending on the parameters, the virus may spread faster through some pathways than others. For example, if *A*_11_ in equation (2.1) is significantly larger than the other matrix elements, then we simply have *R* ≈ *xA*_11_, that is, an infection spread mostly occurs from mildly SARS-CoV-2 infected individuals to result in more mildly infected individuals, and the kinetic parameters associated with mild infection define the *R* value of the whole system:

$$R\approx x\;\frac{1}{\gamma_{1}}\left(\frac{\beta_{1C}b_{C}}{\alpha_{C}}+\frac{\beta_{1H}b_{H}}{\alpha_{H}}\right).$$

On the other hand, if the element *A*_22_ is significantly larger than the rest of the matrix elements, we have *R* ≈ *xA*_22_, and it is COVID-19 severely infected individuals that maintain the epidemic:

$$R\approx x\;\frac{1}{\gamma_{2}}\left(\frac{\beta_{2C}B_{C}}{\alpha_{C}}+\frac{\beta_{2H}B_{H}}{\alpha_{H}}\right).$$

The off-diagonal elements *A*_12_ and *A*_21_ define the contribution of one group of infected to the expansion of the other group. The relative size of these pathways influences the population sizes of *y*_1_ and *y*_2_. For example, *A*_12_ > *A*_21_ tends to increase the population of the mildly infected. The opposite inequality results in the boosting of the *y*_2_ population; see electronic supplementary material for a more precise statement.

Depending on the relative values of the matrix elements *A_ij_*_,_ this model can give rise to a variety of different dynamics and outcomes. To understand this better, it is instructive to first consider extreme cases that bracket all possible outcomes.

— (Ia): Maintenance of the epidemic depends on the mildly infected individuals, *y*_1_, transmitting the virus through the C (or H) compartment and giving rise to mostly more mildly infected individuals. Most infected individuals have mild disease, whereas given subsets develop serious COVID-19.
— (Ib): Maintenance of the epidemic again relies on transmission by *y*_1_ individuals creating more *y*_1_-infected people, but most infected individuals are *y*_2_ and have severe disease.
— (IIa): Maintenance of the infection depends on severely infected individuals, *y*_2_, transmitting the infection (through C or H). The majority of the infected individuals, however, are *y*_1_ and have mild disease, whereas given subsets develop serious COVID-19.
— (IIb): Maintenance of the infection again depends on severely infected individuals, *y*_2_, but most of the infected individuals have severe disease.

**Figure 2.**
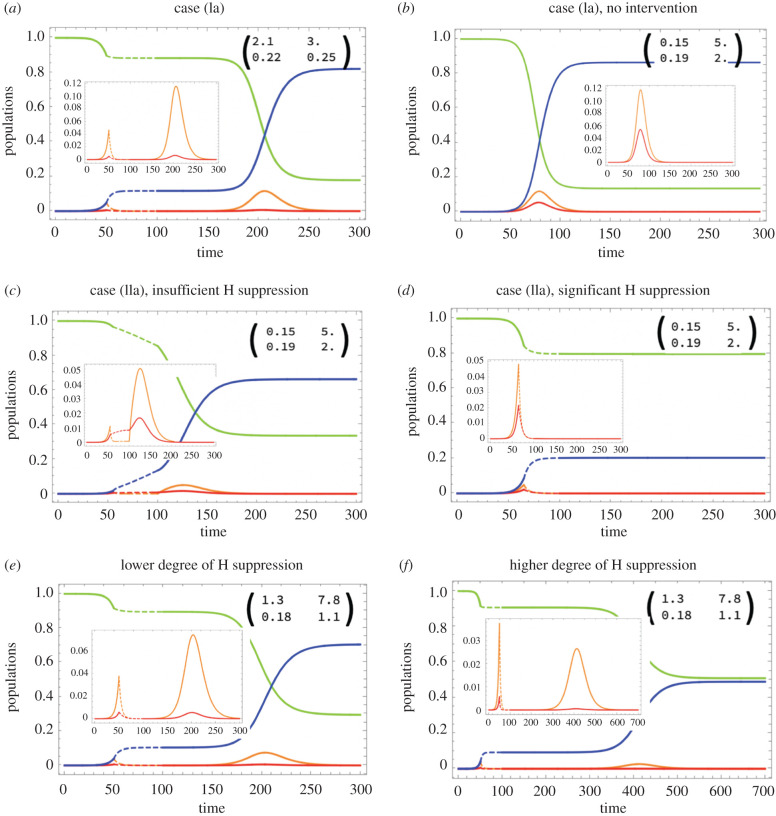
Simulated epidemic dynamics in different cases. Four populations are shown as functions of time: susceptible (green), mildly SARS-CoV-2 infected (orange), severely COVID-19 infected (red) and recovered/dead (blue). Insets show detail in the dynamics of infected, and the matrix elements *A_ij_*. Intervention is shown by dashed lines. It is assumed that during intervention, both H and C channels are suppressed, and afterwards channel C is restored to its full capacity. (*a*) Case (Ia) (see text), where the spread of infection is mostly through mildly infected who comprise the majority; opening up leads to a second wave of infection. (*b–d*) Case (IIa), where the spread of infection is mostly through severely infected although the majority are still mildly infected. In (*b*), no intervention is implemented; the effects of social distancing are shown in (*c*) (insufficient H suppression, a second wave is predicted) and (*d*) (significant H suppression, no second wave). (*e,f*) Both C and H channels contribute about equally to infection spread; opening up the C channel results in a second wave, but a higher degree of H suppression leads to a smaller and more delayed second wave. In all simulations, *R*_0_ = 2.4. Other parameters are given in electronic supplementary material, tables S1–S3. Time-series of the effective reproductive number, *R*_eff_, are shown in electronic supplementary material, figure S3.

While not all of these cases are realistic, an important and novel point emerges from this analysis: under certain assumptions, it is possible that the group of infected individuals that is responsible for maintaining the epidemic (i.e. for keeping *R*_0_ > 1) comprises only a small subset of the infected people. For example, in case (IIa), severely infected individuals that transmit the virus to generate new patients with severe infection, including serious COVID-19 (via hot zone transmission) is critical for keeping *R*_0_ > 1. At the same time, however, the majority of the individuals is mildly infected, *y*_1_, figure 2*b*. This gives rise to the possibility that the targeting of a relatively small fraction of infected individuals in hot zones through specific interventions could curb overall SARS-CoV-2 prevalence in the community at large.

Regime (IIa) is observed if two inequalities hold involving elements of the matrix *A* (see electronic supplementary material):

$$A_{11}<A_{22},\;\mathrm{and}\;\gamma_{1}(A_{11}+A_{12}-1)>\gamma_{2}(A_{22}+A_{21}-1).$$

These conditions are for example consistent with a larger rate of virus deposition by individuals with severe symptoms compared with individuals with mild symptoms (*B_C_*, *B_H_* ≫ *b_C_*, *b_H_*).

### 2.3. Simulating non-pharmaceutical interventions and reopenings

Some valuable insights can be gained by simulating the implementation of non-pharmaceutical interventions and their relaxation. At time *t*_1_, social distancing is initiated by parameter changes that promote a reduction in the reproduction number, e.g. by reducing virus deposition rates (*b_C_*, *b_H_*, *B_C_*, and *B_H_*) and infection rates (*β*_1C_, *β*_2H_, *β*_1H_ and *β*_2C_), or by increasing virus removal rates (*α*_*C*_,*α*_*H*_) and patient removal rates through quarantining (*γ*_1_,*γ*_2_) (figure 1). At time *t*_2_, re-opening is simulated by reverting most parameters back to their original values, with the exception of select parameters connected to either C or H transmission.

We focus on perhaps more realistic model parameter regimes where mild infections predominate. For non-pharmaceutical interventions, we simulate the stay home orders that were implemented around March–April 2020, where it is assumed that virus transmission in the community at large is significantly suppressed, but that hot zone transmission may or may not continue to operate. When simulating the opening of society, different assumptions are made about the extent to which virus transmission resumes in the community at large and in the hot zones.

#### 2.3.1. Community at large transmission alone maintains infection spread

First, we assume that maintenance of the epidemic depends only on community spread and that hot zone transmission contributes much less (case (Ia) above). This corresponds to a scenario in many previously published COVID-19 models (e.g. [13]). Under this assumption, we observe that the suppression of virus transmission in the community at large leads to a marked reduction in infection prevalence. Upon reopening, a pronounced second wave of infection ensues until a vast majority of individuals in the population have been affected (figure 2*a*).

#### 2.3.2. Hot zone transmission alone maintains infection spread

Next, we assume that maintenance of the epidemic relies on hot zone transmission and that community transmission contributes less (case (IIa) above). We distinguish between two scenarios. First, we assume that during stay home orders, hot zone transmission continues to occur. We then assume that hot zone transmission is also suppressed.

(i) Assume that high viral load hot zone transmission remains elevated in certain pockets during the interventions, such that the overall reproduction number is slightly larger than one. In this case, the infection continues to spread slowly during social distancing, and the majority of the infections are predicted to be mild. Under this scenario, infection prevalence is predicted to not decline much during the lockdown, and a renewed and accelerated spread is always predicted to occur upon reopening. Because the reproduction number continues to be larger than one during the intervention period, it can only increase further during the relaxation of the interventions (figure 2*c*).
(ii) If hot zone transmission is also suppressed during the stay home interventions, virus prevalence markedly declines during the intervention period (figure 2*d*). If reopening only leads to a resumption of virus transmission in the community at large while hot zone transmission remains suppressed, no second wave is predicted to occur because the overall reproduction number remains below one (figure 2*d*). If, however, hot zone transmission increases after reopening (due to re-activation of previous hot zones or generation of new ones), the reproduction number can increase beyond one, and a second wave happens in the model (not shown).

#### 2.3.3. Both hot zones and the community at large can maintain virus spread

Here, we assume a more balanced contribution of hot zones and the community at large: either pathway alone can lead to sufficient transmission such that *R* > 1, but both transmission pathways are needed for the virus to achieve its full spread potential. Different outcomes are possible depending on the particulars. For stay home interventions to lead to a marked reduction of infection levels, transmission reduction has to occur both in the community at large and in the hot zones. If hot zone transmission is not significantly affected by the intervention, infection spread will be slowed, but no decline will occur (not shown). In any case, reopening is likely to lead to a second wave, even if virus transmission only resumes in the community at large. The magnitude and timing of the second wave depend on social distancing and reopening parameters. For example, if hot zone transmission is suppressed to a larger degree, the second wave will be characterized by a slower growth rate and a lower size (figure 2*f* compared to figure 2*e*; note the different range of the horizontal axes and the lower final epidemic size).

### 2.4. Which parameters should be targeted?

Our methodology allows us to calculate the effective reproductive number, *R*_eff_, which indicates whether the infection (in a deterministic model) will increase or decrease at a given point of the epidemic. The effective reproductive number is given by *R*_eff_ = *xr* (equation (22) of the electronic supplementary material), where *x* is the number of susceptible individuals and the full expression for *r* is given in equation (21) of the electronic supplementary material.

We performed a sensitivity analysis of the parameter *r* with respect to all the parameters in regime (IIa), since this regime is characterized by interesting dynamics that go beyond those observed in simpler SIR models. The results are shown in electronic supplementary material, figure S4. Although *r* depends in non-trivial ways on all 12 parameters or rates in the model, there are four parameters that have markedly higher influence on the effective reproductive number. These parameters are (in regime (IIa)):

*B*_H_ (virus deposit rate from severely infected individuals in the hot zones)
*α*_H_ (virus inactivation/removal rate in hot zones)
*β*_2H_ (probability to become severely infected through a hot zone transmission)
*γ*_2_ (recovery/removal rate of infected individual with severe disease).

The sensitivity of the effective reproductive number to these parameters is orders of magnitude higher than that of the other parameters in the system. Interestingly, all of these parameters are related to hot zone/severe infection transmission channel.

This analysis has direct implications for suggesting the most effective strategies of reducing infection spread in the context of regime (IIa). Some of the most effective measures include: reducing the deposition of the virus in hot zones by restricting access of severely infected individuals (reducing *B*_*H*_), increasing viral removal rate in the hot zones by surface disinfection/aeration (increasing *α*_*H*_), reducing hot zone transmission by, for example, social distancing and enforcing wearing face covers, as well as staggering shifts of essential workers in workplaces (decreasing *β*_2H_) and quarantining/isolation of symptomatic individuals (reducing *γ*_2_).

### 2.5. Further complexities

**Figure 3.**
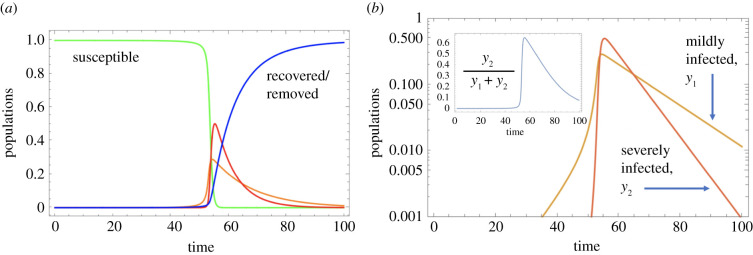
More realistic scenarios of infection spread: included is a saturation term in the C and H channels, and the probability of becoming severely infected through channels C and H depends on the infection level, resulting in a change in the proportion of severely infected. The epidemic dynamics are shown in (*a*) and the fraction *y*_2_/(*y*_1_ + *y*_2_) in the inset in (*b*). For all parameter values, see electronic supplementary material, table S4.

Additional insights can be obtained by incorporating further complexities that might better characterize the SARS-CoV-2/COVID-19 pandemic. A mathematical model that includes these assumptions is presented in the electronic supplementary material. In the example of figure 3, we assume that once the virus load in the hot zones rises to high levels, it is likely that the rate of virus transmission saturates rather than increases in an unlimited way. Further, we assume that the severity of infection is not only transmission zone-dependent, but that a higher viral load in both C and H locations results in a higher chance of severe infection. The model now predicts that the fraction of severe infections rises as total infection levels increase, but that it then declines post-peak (figure 3*b*). This might correspond to the observations of increased disease severity as the epidemic expands, and then reduced disease severity as the outbreak declines, which has been observed in Italy [36] and Sweden [37], among other locations. Importantly, the simulations in figure 3 show that during the initial stages of spread, mild SARS-CoV-2 infections predominate, but once the infection has spread beyond a certain threshold, the total viral load in both hot zones and the community at large increases to the point that severe COVID-19 cases predominate. This would translate into a health crisis with an overload of the health systems and into high levels of mortality even if healthcare resources did not become limiting. According to this model, it is critical to implement interventions sufficiently early rather than only once increased mortality becomes apparent (which occurs with an additional delay).

## 3. Discussion

We have presented and analysed a new epidemiological model in which infection severity depends on the virus dose received during transmission, assuming that exposure to higher virus loads is more likely to occur under certain conditions, which we refer to as transmission hot zones. These high viral loads can be reached by silent, undetected amplification cycles before the first clinical cases are detected [33]. All model assumptions are rooted in biological/epidemiological data. (i) With other coronaviruses, and with SARS-CoV-2, it has been established that infection dose influences disease severity [26–29]; the model presented includes this key assumption. (ii) Data clearly indicate that virus transmission is more effective in certain settings (where exposure to viral load is higher) than in others [18,19]. These include nursing homes, food processing plants, prisons, restaurants, bars, gyms, etc. This is the basis for including hot zone transmission in our model. As with all mathematical modelling studies, there are uncertainties, and especially with respect to unknown parameter values. Our understanding of the dynamics that characterize this model, however, does not depend on knowledge of precise unknown parameter values, because a comprehensive analytical understanding is gained as a result of this relative ‘simplicity’ of the model. We present possible behaviours along with conditions when they are observed, which enhances our understanding of the possible consequences arising from hot zone transmission.

One important insight was that in one of the model parameter regimes, a relatively small population of severely infected patients can be responsible for driving infection through hot zone transmission, even though the majority of infections are mild and acquired in the community at large. A logical consequence of this is that even though the majority of infections are found in the community at large, interventions that specifically target the hot zones can be very effective. They can reduce the viral reproduction number, inducing a decline of the overall infection prevalence, including that in the community at large. Hence, effective virus suppression in hot zones might allow to maintain a relatively strong degree of overall infection control with fewer restrictions in the community at large, although critical interventions in the community at large will most likely also be required.

More broadly, the model explored here allows for a significant heterogeneity of outcomes which can inform us of possible mechanisms of infection spread in different locations. In contrast with this, a basic SIR model without any additional complexity cannot account for the observed heterogeneity of outcomes. More specifically:

(1) In figure 2*c*, we see a scenario where the community at large is shut down (with the corresponding *R*_0_ < 1), but despite these intervention measures, the infection keeps growing during the lockdown, because the hot zones continue to transmit. This is consistent with the March 2020 lockdown in a variety of states, such as California, when transportation and other measures were significantly down, but the infection continued to spread, albeit at a relatively slow rate. In a simple SIR model with *R*_0_ < 1 during the lockdown, the infection levels will inevitably decline during the intervention period, because this model lacks the hot zone transmission channel, which, even if it comprises only a small minority of cases, could ‘carry’ the infection spread.
(2) In figure 2*d*, we present a scenario where upon the reopening of society, the infection does not come back. This is because after the end of lockdown, although community spread resumes, the hot zone transmission continues to be suppressed, and this is sufficient to keep the overall *R*_0_ less than one. Hence, the epidemic does not experience a second wave absent further changes (such as increases in transmission rates resulting from changes in behaviour or other factors). This is consistent with the observations in the post-lockdown months in some east coast states, like New York, and some European countries in summer 2020. Following the first infection wave, infection levels remained suppressed for several months, despite significant reductions of non-pharmacological interventions in the community during this phase. In a simpler SIR model without added complexity, returning community *R*_0_ to values greater than one will inevitably cause a second wave of infection without delay. Our model further makes the prediction that renewed hot zone transmissions would be able to trigger renewed virus spread. This is again consistent with observations. For example, in Europe, the initial growth in case numbers during early autumn of 2020 seemed to be associated with hot zones, such as food processing plants [20].

While our model can be used to interpret the heterogeneity in epidemiological trajectories during and after the implementation of non-pharmaceutical interventions, it is important to note that other mechanisms could also account for these differences (e.g. social network structure, population demographics, genetics, changes in non-pharmacological and pharmacological interventions, etc.) if they are included into the SIR framework. The model properties discussed here identify transmission hot zones as one mechanism that can contribute to the observed heterogeneity in data, which is a new insight and warrants further epidemiological investigation. The heterogeneity of dynamics observed in the data on SARS-CoV-2 spread appears to be the most consistent with a complex epidemiology modulated by a number of different factors and mechanisms. There is already a growing list of factors known to affect it, to which we add now the consideration of the hot zone spread.

SARS-CoV-2 transmission, as well as past coronavirus outbreaks, have been characterized by large numbers of infections resulting from superspreading events; the corollary to this finding is that most individuals infected with these viruses did not transmit the virus as efficiently as those participating in the superspreading events [18,19,25,38]. The ‘hot zone’ transmission framework proposed here is broadly consistent with these findings: hot zones are characterized by the exposure of susceptible individuals to a relatively high virus dose, which may lead to more severe disease. Superspreading events are a type of hot zone in our model, because they typically refer to one-time larger gatherings of people, including several infected individuals shedding virus at the same time, which can provide the high-dose exposures. Another type of hot zone involves the repeated meeting of the same people at a given location, which can result in the amplification of viral load in that location. While early virus exposure from one infected person would likely constitute a low-dose exposure and result in mild infection in several individuals, these individuals would then all shed virus, thus increasing the viral dose with which others are infected. Hence, amplification through asymptomatic or mild cases will eventually result in exposure to a higher virus dose and in a higher chance of severe disease in the newly infected people, thus generating a hot zone environment. Such characteristics could exist in crowded or poorly ventilated office work spaces, schools, prisons, places of worship, long-term care or assisted living communities and healthcare or hospital settings, as well as in multigenerational households, which have all been identified as transmission clusters in various countries [18,19]. Long-term care facilities and hospitals might be especially prone to this effect, which is consistent with the large percentage of deaths in long-term care facilities. People with pre-existing health conditions regularly visit hospitals, which could start such an amplification cycle anywhere in a hospital before the presence of the virus in the location is known.

These notions are consistent with data from Italy, where at least some hospitals were identified as a major contributor to early COVID-19 spread, like in Bergamo [39]. Similarly, in the United States, an infection cluster was identified in a hospital in Boston [40]. These notions are further consistent with the many analyses of the disproportionately high mortality of elderly people residing in long-term care living facilities [41,42] rather than at individual homes across the world [43], and with the disproportionate impact on minority communities in America [44], who may have been required to physically be at the workplace during the outbreak due to their employment in ‘essential services’ like transportation and food preparation. A number of Asian and Pacific countries implemented strong protection measures in the healthcare system before the first COVID-19 cases were identified, due to previous experiences with SARS and MERS. Countries such as South Korea performed intensive targeted testing, resulting in early identification of infected individuals, which were thus removed from both transmission chains and any potential amplification circles [45]. Such strategies limit the seeding of transmission hot zones and prevent the initial rounds of viral load amplification, which might have contributed to the relatively lighter disease burdens documented there, despite somewhat less strict social distancing [46].

These considerations also provide a motivation to act early and decisively to prevent the amplification of the viral loads and to prevent potential transmission hot zones with severe disease from forming. Facemasks might be crucial in this respect, because they reduce exposure dose. Facemasks can thus turn a potential hot zone that drives infection spread into a lower dose transmission environment, which does not have the same ability to maintain overall infection spread.

This model can be further refined by including risk factors, social networks and other complexities, which all are likely to be critical for any practical predictive use. For example, we assumed in our model that disease severity was associated with the dose of virus exposure. Risk factors (importantly age and comorbidities), however, are a well-known important determinant of disease severity, and incorporation of these additional parameters will refine the accuracy of the model. Moreover, model predictions depend on assumptions, which need to be tested with epidemiological, clinical and virology data. The strength of modelling, however, is to identify potential key drivers of the pandemic, which we would not be aware of otherwise, thus directing the required epidemiological, clinical and virology investigations. The model suggestion that severe infection transmission through high viral load exposure in hot zones might be an important driver of SARS-CoV-2 spread, even as mild disease cases predominate, could allow us to improve the outcome of reopening society through targeted non-pharmaceutical interventions or preferentially targeting vaccines to those participating in hot zone transmission situations.

## 4. Materials and methods

We have modelled the spread of infection by using ordinary differential equations (ODEs) of SIR type, where we distinguished between patients with a mild or almost asymptomatic infection and those with severe SARS-CoV-2 infection. Transmission happened through two different ‘channels’. Several extensions of the model are also considered including a model with saturation in the infection term. The precise formulations and complete analysis of the ODEs are presented in the electronic supplementary material.

## Data accessibility

This article has no additional data.
